# A 28-Year-Old Woman with Fever, Weight Loss, Pancytopenia, and Multiple Hepatosplenic and Bone Lesions

**DOI:** 10.1155/2015/759856

**Published:** 2015-07-13

**Authors:** Fereshte Sheybani, Bezat Amiri, HamidReza Naderi, Mohammad Reza Sarvghad

**Affiliations:** Department of Infectious Diseases, Faculty of Medicine, Mashhad University of Medical Sciences, Iran

## Abstract

Brucellosis is a systemic zoonotic infection that can involve any organ or system in the body. It may present with a broad spectrum of clinical manifestations. Considering such myriad presentations, brucellosis should always be considered in the differential diagnosis of any febrile illness in a compatible epidemiological context. Here, we report a rare presentation of acute brucellosis with multifocal osteomyelitis and hepatosplenic lesions.

## 1. Case Report 

**Figure 1:**
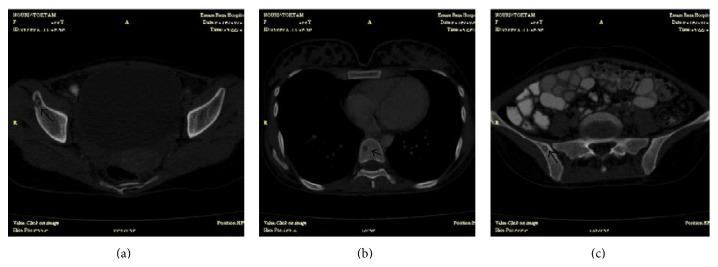
Computed tomography scan shows multiple small osteolytic lesions containing a sequestrum in acetabular roof (a), 8th thoracic vertebra (b), and ilium (c).

**Figure 2:**
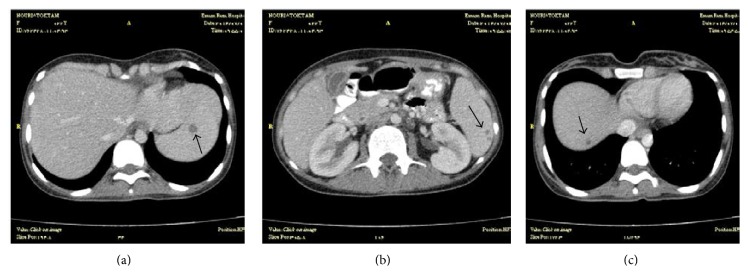
Computed tomography of the abdomen and pelvis with intravenous and oral contrast reveals multiple hypodense lesions in spleen ((a) and (b)) and liver (c).

A 28-year-old woman, a homemaker, presented with 3-month history of fever, shaking chills, sweats, malaise, anorexia, severe weight loss up to 15 kilograms, and nausea and vomiting. She lived in a village near Mashhad city of Iran, in a* livestock-keeping family,* and was breastfeeding her healthy infant 8 months ago. On physical examination, the patient had a blood pressure of 105/60 mmHg, oral temperature of 39.5°C, heart rate of 110 beats/min, and respiratory rate of 24/min, and the findings of a neuropsychiatric examination were significant for depressed mood and psychomotor retardation out of the proportion to the severity of other symptoms as a symptom of depression. Physical examination was otherwise unremarkable, except for mild hepatosplenomegaly. There were no mucocutaneous lesions, peripheral adenopathies, or cardiac murmurs. Laboratory examination revealed pancytopenia (hemoglobin 8.8 g/dL (normal, 12.30–15.30 mg/dL), white blood cell counts 2.2 × 10^9^ cells/L (normal, 4.4–11.3 × 10^9^ cells/L) with 91% neutrophils, and platelet counts 120 × 10^9^ cells/L (normal, 150–450 × 10^9^ cells/L)), elevated liver transaminases (aspartate aminotransferase 239 U/L (normal, 5–40 U/L), alanine aminotransferase 113 U/L (normal, 5–40 U/L)), elevated serum alkaline phosphatase of 1194 IU/L (normal, 64–306 U/L), elevated lactate dehydrogenase of 988 U/L (normal, 100–500 U/L), and normal gamma-glutamyltransferase. Screening antibody test for human immunodeficiency virus was negative. Hepatitis B surface antigen, antibody to hepatitis B core antigen, anti-hepatitis C virus, and anti-nuclear antibody were negative. Serum ferritin level was 1108 ng/mL (normal, 12–150 ng/mL), reticulocyte counts were 0.5% (normal, 0.5–2%), and ESR was 26 mm/h (normal, 0–20 mm/h). High-resolution computed tomography of the lung and computed tomography of the abdomen and pelvis with intravenous and oral contrast were performed that revealed multiple small osteolytic lesions containing a sequestrum in the acetabular roof, ilium, and 8th thoracic and 2nd lumbar vertebrae (Figure 1), multiple hypodense lesions in liver and spleen (Figure 2), and multiple para-aortic lymphadenopathies.

**Figure 3:**
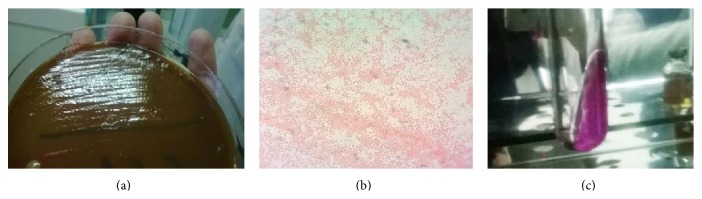
*Brucella* colonies on blood agar (a); Gram stains of colonies revealing Gram-negative coccobacilli (b); positive urease test (c).

Based on clinical and para-clinical findings, presumptive diagnosis of lymphoproliferative disorder, hemophagocytic syndrome, or metastatic carcinoma was made. Bone marrow aspiration result was inconclusive but did not rule out a lymphoproliferative disease. A bone marrow biopsy specimen was also unremarkable with no granuloma or evidence of malignant or abnormal hemophagocytic cells. Echocardiography was normal. After seven days of incubation,* Brucella melitensis* was isolated from the patient's blood culture and appeared as Gram-negative coccobacilli with positive urease and oxidase tests (Figure 3). Based on the recommended treatment options of complex or focal brucellosis [1], the patient was treated with Doxycycline and Trimethoprim-Sulfamethoxazole for four months and Gentamicin for initial three weeks with complete recovery of symptoms and signs. Serial ultrasonography and end-of-treatment computed tomography scan of the abdomen revealed disappearance of hepatosplenic lesions. The patient had no recurrence and remained healthy during the 15-month follow-up.

## 2. Discussion 

This case features some of the rare manifestations of brucellosis such as multifocal osteomyelitis and hepatosplenic lesions concurrently. Because of confusing clinical presentation, she was initially misdiagnosed as a case of lymphoproliferative disorder or metastatic carcinoma.

Brucellosis is a systemic zoonotic infectious disease caused by Gram-negative bacilli of the genus* Brucella*. Although the disease is prevalent worldwide, it is particularly endemic in many Middle Eastern countries, the Mediterranean region, and the Arabian Peninsula [2]. Brucellosis is a public health problem in many developing countries, including Iran. In these regions brucellosis has primarily been caused by* Brucella melitensis*, which is the most virulent species, and causes the most severe acute forms of brucellosis with disabling complications [3]. Brucellosis can involve any organ or system of the body. When involvement of a specific organ predominates, the disease is often termed focal or localized [4]. Focal forms of brucellosis may occur in up to a third of patients and as they are considered true complications of the disease, they tend to have a worse prognosis compared with the nonfocal forms. These focal forms may include osteoarticular, neurological, genitourinary, liver, hematological, and cardiac involvements [2].* Brucella* bacteremia can also cause abscess formation in the spleen, liver, or other organs [5]. Nevertheless, some forms of focal involvement such as hepatosplenic abscesses and multifocal or extraspinal osteomyelitis have rarely been seen and they are quite uncommon. These rare manifestations can sometimes mislead a clinician to establish a diagnosis of brucellosis and they might contribute to the misdirected diagnoses even in geographical areas that are endemic for this zoonosis.

Osteoarticular involvement is the most frequent focal involvement of brucellosis. It has a wide range of presentation, including arthritis, spondylitis, bursitis, tenosynovitis, and osteomyelitis [3, 6, 7]. Sacroiliitis is the most common complication, followed by spondylitis and peripheral arthritis [8].* Brucella* osteomyelitis may appear as a radiolucent area and it may be confused with tumor lesion on radiographs [6]. Although multiple articular involvements have been reported in 17% of patients with brucellosis [7], we found few reports of multifocal osteomyelitis as a manifestation of brucellosis [8]. There are also relatively few reports of brucellar osteomyelitis of the extraspinal skeleton in literature. The reported case had these two rare manifestations simultaneously. She has multifocal osteomyelitis involving acetabular roof, ilium, and thoracic and lumbar vertebrae. We found only one report of acetabular brucellar osteomyelitis in English literature [6].

Our report also described a rare presentation of acute brucellosis with bacteremia and multiple hepatosplenic lesions. Although hepatomegaly and splenomegaly are frequent findings on physical examination, a more unusual complication of human brucellosis is the development of abscesses of the liver and spleen [5]. In a study by Colmenero et al. hepatosplenic abscesses were detected in 0.8% of 805 patients [9]. The clinical features in patients with this complication of brucellosis have ranged from fever of unknown origin to recurrent or chronically draining cutaneous sinuses. In most patients disease was localized to the liver and/or spleen at the time of diagnosis, but several had concurrent involvement of bones or lymph nodes [10]. Yilmaz et al. [2] reviewed the English language literature of the last 50 years and found 20 adult cases with splenic abscess(es) caused by* Brucella* species of whom 70% were due to* B. melitensis*. In Turkey, Pourbagher et al. [11] reviewed 251 cases of brucellosis and identified splenic abscess in 1.6% of cases with an ultrasound. Other rare complications associated with splenic involvement in brucellosis include splenic infarction, [12, 13], splenic subcapsular hematoma, and spontaneous splenic rupture [5]. Most patients with hepatic and splenic abscess due to brucellosis have chronic infection. Chronic hepatic and splenic involvement is characterized by tissue calcification. Only few reports have described the development of splenic abscesses during the course of acute brucellosis [14].

Accurate diagnosis of brucellosis in any species is generally fairly straightforward but may be very difficult in some cases. The only definite diagnosis, the “gold standard,” is the recovery of the causative agent from blood, bone marrow, or other tissues [15]. A 97% yield of* Brucella* species isolate from bone marrow cultures and 83% yield of* Brucella* species isolate from blood cultures for patients with acute brucellosis have been reported. The yield of blood cultures decreased significantly in association with subacute infection (40%) and chronic infections (25%) [10]. Isolation of* Brucella* species requires highly skilled personnel and an extended turnaround time for results and it is considered a hazardous procedure. Hence, brucellosis is generally diagnosed by detection of an elevated level of antibody in serum or body fluid. This is a presumptive diagnosis as other microorganisms [15].

The mortality rate of splenic abscess is reported to be 100% without treatment [16]. The prognosis of splenic abscess(es) is good with early diagnosis and treatment [17]. Although the therapy for uncomplicated brucellosis is well established, the best regimen for the treatment of localized lesions has not been clearly defined. Successful treatment of liver and splenic abscesses in most reported cases has required the combination of surgery and antimicrobial therapy. The treatment of splenic abscess with antibiotics alone has been reported; however this modality appears to be successful only in the early stages of the diseases when there is no calcification in the lesions [2]. A therapeutic approach in the early stages, with the use of antibiotic therapy alone or associated with nonsurgical drainage, may be an initial option but prolonged therapy over several months may be required and careful follow-up is essential because the complete cure of the disease cannot be guaranteed. Prolonged medical treatment in combination with serial abdominal ultrasonography can increase the chance of cure without surgical intervention in* Brucella* splenic abscess. Surgical treatment must be considered in patients with splenic abscess who do not respond to antibiotic treatment [14]. In the study of Colmenero et al., therapeutic failure was defined as the lack of regression in the lesions after appropriate antibacterial treatment for two months [9]. The treatment of chronic lesions should involve a combination of medical and surgical therapy [2]. In the presence of hepatosplenic abscess related to chronic brucellosis, medical therapy alone has achieved a good early response in only 20–40% of cases. Duration of the medical therapy must not to be less than 6 weeks [14].

In conclusion, human brucellosis is characterized with unbelievably vast and complex spectrum of clinical manifestations and one should think about this disease even in unspecific cases like this, especially in endemic regions such as Iran.
